# Increased Stiffness of the Superficial Cervical Extensor Muscles in Patients With Cervicogenic Headache: A Study Using Shear Wave Elastography

**DOI:** 10.3389/fneur.2022.874643

**Published:** 2022-05-27

**Authors:** Li-Zhen Lin, Yan-Ni Yu, Jie-Cheng Fan, Pei-Wu Guo, Chun-Feng Xia, Xue Geng, Shu-Yun Zhang, Xiang-Zhen Yuan

**Affiliations:** ^1^Department of Rehabilitation Medicine and Physical Therapy, Faculty of Rehabilitation Medicine, Weifang Medical University, Weifang, China; ^2^Department of Ultrasound, Weifang People's Hospital, Weifang, China; ^3^Department of Rehabilitation Medicine, Weifang People's Hospital, Weifang, China; ^4^Department of Neurology, Weifang People's Hospital, Weifang, China

**Keywords:** cervicogenic headache, superficial cervical extensor muscles, stiffness, elasticity, shear wave elastography

## Abstract

**Background:**

Cervicogenic headache (CEH) is a secondary headache caused by lesions of the cervical spine and surrounding soft tissues. Cervical muscle dysfunction may be related to the onset of CEH. However, whether cervical muscle stiffness changes in patients with CEH has not been well studied. The purpose of this study was to explore changes in superficial cervical extensor muscle stiffness in patients with CEH using shear wave elastography (SWE).

**Methods:**

In this study, 19 patients with CEH and 20 healthy controls were recruited. Superficial cervical extensor muscle stiffness was obtained from SWE, and the SuperLinear SL10-2 MHz linear array probe in the musculoskeletal muscle mode was chosen as the transducer. Regions of interest in the trapezius (TRAP), splenius capitis (SPL), semispinalis capitis (SCap), and semispinalis cervicis (SCer) were manually segmented. Correlations between superficial cervical extensor muscle stiffness and visual analog scale (VAS) scores, age, and body mass index (BMI) were analyzed using Pearson's correlation. Receiver operating characteristic (ROC) curve was used to investigate the diagnostic ability of superficial cervical extensor stiffness for CEH.

**Results:**

Superficial cervical extensor muscle stiffness on the headache side of patients with CEH was higher than that on the non-headache side and in healthy controls (*p* < 0.05). Increased stiffness was also observed in SCer on the non-headache side of patients with CEH compared to healthy controls (*p* < 0.01). In patients with CEH, SCer stiffness was positively correlated with VAS scores (*r* = 0.481, *p* = 0.037), but no correlation was found between other muscles and VAS scores (*p* > 0.05). The areas under the curve of TRAP, SPL, SCap, and SCer in diagnosing CEH were 0.766, 0.759, 0.964, and 1.000, respectively.

**Conclusions:**

Increased stiffness was observed in the superficial cervical extensor muscles on the headache side of patients with CEH. SCer stiffness was correlated with headache intensity in patients with CEH and may provide clues for the diagnosis of CEH.

## Introduction

Cervicogenic headache (CEH) is a common secondary headache caused by lesions of the cervical spine and surrounding soft tissues. The prevalence of CEH has been reported to be 0.4–2.5% in the general population (1) and as high as 15–20% in patients with chronic headache (2). CEH is characterized by a unilateral headache, and pain that begins in the neck or occipital region can gradually spread to other regions of the face and head, mainly in the temporal and frontal regions. A small number of patients may feel discomfortable in ears and alar of nose, vertigo, and nausea. Neck musculoskeletal dysfunction may be a potential cause of CEH (3), and the craniocervical flexion test revealed deep cervical flexor muscle dysfunction in patients with CEH (4). Ultrasound examination showed that the size and thickness of the anterior and posterior cervical muscles changed in patients with CEH, accompanied by fat infiltration and muscular atrophy (5, 6). These pathological changes were observed in the semispinalis capitis (SCap), longissimus capitis (3), longus colli, and suboccipital muscles in patients with CEH (7). About 93% of patients with CEH have increased cervical muscle tension and stiffness (8), which may induce secondary muscle spasm and neck pain. Currently, it is unclear how cervical extensor muscle stiffness is altered in patients with CEH. In addition, there are few simple and effective examinations for the clinical diagnosis of CEH.

Lesions of the cervical extensor muscles may be related to the onset of CEH. The cervical extensor muscles are the basis for maintaining normal function of the cervical vertebra. The cervical extensor muscles include the trapezius (TRAP), splenius capitis (SPL), SCap, semispinalis cervicis (SCer), and multifidus (9) from the superficial to deep layer, with the function of functional extension, ipsilateral side-bending, and contralateral rotation of the head and neck. The muscular control of posture suggests that muscle tension has a supportive effect (10, 11), while increased muscle tension can limit movement (12, 13). In patients with CEH, the limited range of motion of the cervical vertebra (especially, the rotation of C1 and C2) and persistent poor posture (14) may lead to long-term changes in head and cervical muscle work, as well as changes in muscle structure and elasticity. Therefore, we hypothesized that cervical extensor muscle stiffness might change with the progression of CEH.

Shear wave elastography (SWE) is a non-invasive, quantitative, and real-time imaging of soft tissue stiffness, which is considered to be more objective and reproducible than compression sonoelastography (15). SWE uses an acoustic radiation force pulse to generate shear waves that propagate perpendicular to the acoustic beam, causing transient displacements (16). The shear wave velocity at each pixel is directly related to the shear elastic modulus (stiffness), which is an absolute measure of tissue elasticity (15, 17). SWE is an effective method to quantify changes in muscle stiffness (18). Recent studies have demonstrated that SWE can effectively measure the elasticity of the cervical extensor muscles in both relaxed and contracted states (19–21).

In this study, SWE was used to study superficial cervical extensor muscle stiffness in patients with CEH and in healthy controls. The shear elastic modulus of TRAP, SPL, SCap, and SCer was quantitatively measured. In addition, we investigated the factors that may be associated with superficial cervical extensor muscle stiffness and explored the diagnostic ability of superficial cervical extensor stiffness for CEH.

## Materials and Methods

### Subjects

A total of 19 patients with unilateral CEH were recruited for this study from the Department of Rehabilitation Medicine of Weifang People's Hospital between June 2021 and October 2021, and another 20 healthy volunteers with matched age and gender were recruited as a control group. Patients with CEH were diagnosed by a neurologist (SYZ) with more than 30 years of experience. This study was approved by the Local Ethics Committee, and all patients were fully explained about this study and were given informed consent.

Inclusion criteria for patients with CEH were based on the International Classification of Headache Disorders-3 (ICHD-3). These criteria included clinical or imaging evidence of a lesion within the cervical spine or soft tissues of the neck, known to cause headache. Any headache fulfilling the following standards or evidence of causation was demonstrated by at least two of the following: firstly, the headache has a temporal relationship to the onset of the cervical disorder; secondly, the headache can be significantly relieved as the cervical spondylosis improves or subsides; thirdly, the cervical range of motion is reduced and the headache is significantly worsened by provocative maneuvers; and fourthly, the headache is abolished following diagnostic blockade of a cervical structure or its nerve supply. Patients with CEH aged 16–55 years without cognitive impairment were included in this study. Exclusion criteria included combination with other types of headaches, a previous history of severe head and cervical trauma or cervical surgery, cervical congenital malformation, osteoporosis, cervical vertebra fracture, bone destruction, patients with mental or psychological disorders unable to cooperate with the examination, pregnant women, and patients after treatment such as massage, physiotherapy, and muscle relaxants.

Inclusion criteria for healthy controls were 16–55 years of age, with no history of CEH and cognitive impairment. Exclusion criteria included other types of headaches, a previous history of severe head and cervical trauma or cervical surgery, cervical congenital malformation, and pregnancy.

### Visual Analog Scale Assessment

Before measuring stiffness, we measured the headache intensity with the visual analog scale (VAS) (22) in the CEH group. The level of pain ranges from 0–10, where 0 indicates no pain and 10 means extreme pain. The VAS scores were assessed based on the subjective pain intensity of patients with CEH by a rehabilitation physician (LZL) on the day of ultrasound examination.

### SWE Data Acquisition

The subjects were lying prone, with their shoulders in abduction and external rotation, hands overlapped under the forehead and the chest supported by a pillow to make the neck in a neutral position. The stiffness of the muscles on the left and right sides of the back of each subject's neck was measured. Muscle stiffness was measured by shear wave ultrasound elastography (Supersonic Imagine, Aix-en-Provence, France), and a SL10-2 MHz linear array probe in the musculoskeletal muscle mode was chosen as the transducer, with a shear elastic modulus up to 180 kPa. Ultrasound was performed by a sonologist (YNY) who had 5 years of experience in musculoskeletal ultrasound and was blinded to the information of subjects. The measured shear elastic modulus values were expressed in kPa, the larger the shear elastic modulus value, and the greater the muscle stiffness.

**Figure 1:**
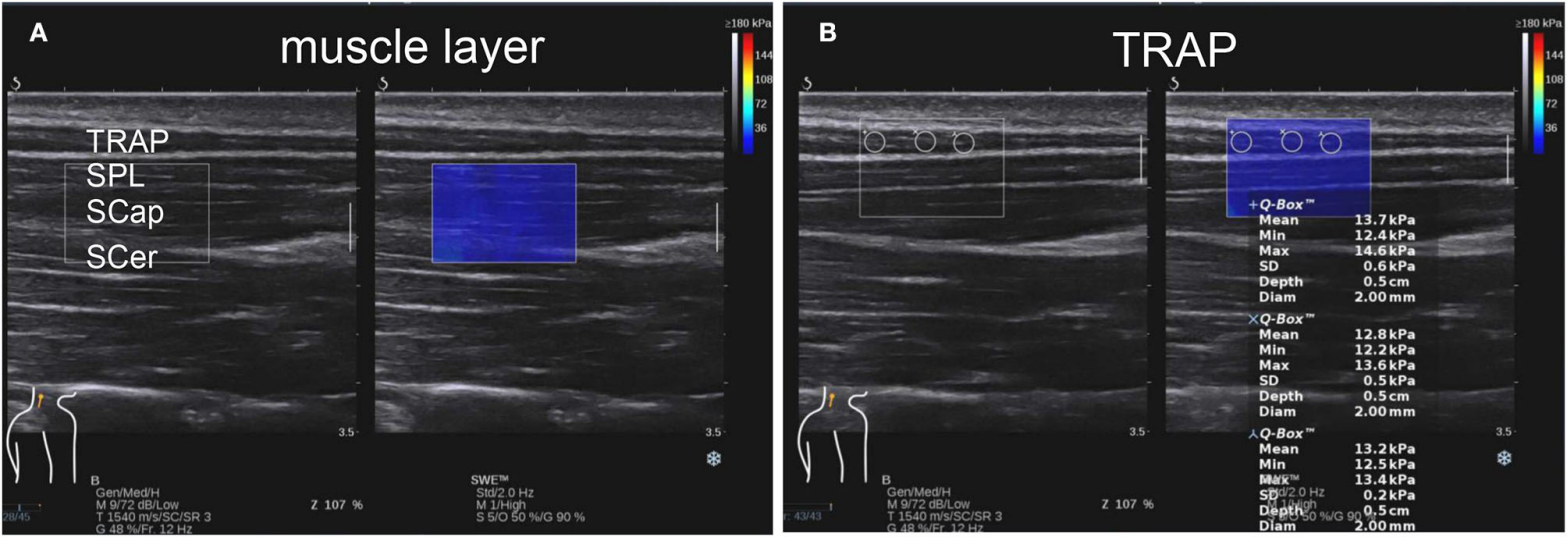
In the shear wave elastography (SWE) mode, image **(A)** showing muscle stratification of TRAP, splenius capitis (SPL), semispinalis capitis (SCap), and semispinalis cervicis (SCer) on the left, while the dark blue area on the right is the elastic sampling frame. Image **(B)** shows representative elastic diagrams of TRAP of the cervicogenic headache (CEH) group. The shear elastic modulus of region of interest (ROIs; the three white circles) in the elastic sampling frame is calculated. Three ROI values of TRAP are 13.7, 12.8, and 13.2 kpa. TRAP, trapezius.

Superficial cervical extensor muscle stiffness was measured at 2 cm lateral to the spine around the fourth cervical vertebra (19), and the probe was placed on the skin with minimal pressure in all participants (23). Given the anisotropy of the ultrasound muscle tissue, B-mode ultrasound was first used to identify the shape of the muscle. Then, the probe was put longitudinally, parallel to the measured muscle, and switched to SWE mode (Figure 1 and Supplementary Figure S1). Each muscle was maintained in SWE for 5–6 s to obtain a clear elastography map. After filling the sampling frame, each muscle was measured with a 2-mm-diameter region of interest (ROI). SWE could automatically calculate the shear elastic modulus (μ) within the ROI according to the formula μ = ρ*v*2, where ρ is the muscle density and *v* is the speed of the shear wave propagating in the muscle tissue (24). ROIs were measured for each muscle in the elastic frame and the mean values of muscle stiffness were calculated. To ensure the accuracy of the measurement data, we repeated the measurement three times and took the mean values of three measurements. We measured superficial cervical extensor muscle stiffness on the headache and non-headache side in patients with CEH. In healthy controls, superficial cervical extensor muscle stiffness on the right and left side was also measured and their mean values were used for statistical analysis.

### Statistical Analyses

In this study, all statistics were performed with Statistical Product and Service Solutions (SPSS, version 26.0). Numerical data were presented as mean ± standard deviation (SD), and the Shapiro–Wilk test was used for normality test. One-way analysis of variance (ANOVA) was used for comparison of muscle stiffness between the different groups, and the least significant difference (LSD) test was used for *post hoc* multiple comparisons (Tamhane's T2 test for unequal variances). Independent samples *t*-test was used to compare age and BMI between patients with CEH and healthy controls. Correlations between cervical extensor muscle stiffness and VAS scores, age, and body mass index (BMI) were analyzed using Pearson's correlation. The diagnostic ability of the stiffness of TRAP, SPL, SCap, and SCer for CEH were determined by performing receiver operating characteristic (ROC) curve analysis. A two-tailed *p* < 0.05 was considered significant.

## Results

### Subject Characteristics

A total of 19 patients with CEH (9 men and 10 women) and 20 age-matched healthy controls (10 men and 10 women) were included in the analysis. The mean age of patients with CEH and healthy controls was 27.47 ± 8.38 (range 16–44) years and 24.60 ± 5.92 (range 16–40) years, respectively. The mean BMI of patients with CEH and healthy controls was 21.75 ± 2.65 (range 17.3–26.8) and 21.63 ± 2.74 (range 17.6–25.8), respectively. There were no statistically differences between the two groups for age (*p* = 0.227) and BMI (*p* = 0.888). The mean VAS score in patients with CEH was 5.21 ± 1.13 (range 3–7) (Table 1).

**Table 1:** Subject characteristics.

	**Patients with CEH**	**Healthy controls**	***P*-value**
	***N* = 19**	***N* = 20**	
Male/female	9/10	10/10	1.000
Age (year)	27.47 ± 8.38	24.60 ± 5.92	0.227
BMI (kg/m2)	21.75 ± 2.65	21.63 ± 2.74	0.888
VAS scores	5.21 ± 1.13	−	−

*VAS, visual analog scale; BMI, body mass index; CEH, cervicogenic headache; N, number*.

*p < 0.05 was considered significant*.

### Comparison of Stiffness

**Figure 2:**
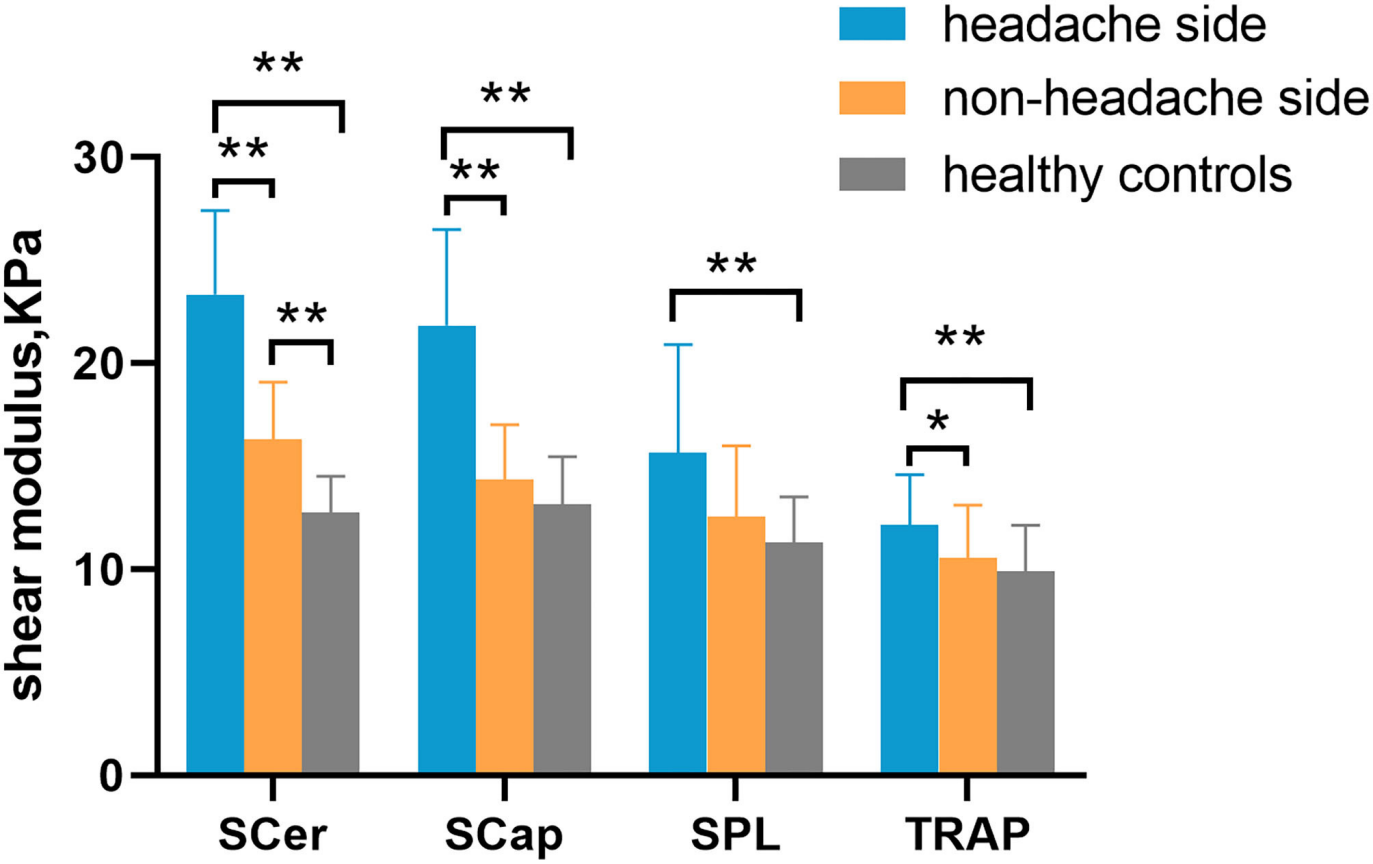
Comparison of superficial cervical extensor stiffness between patients with CEH and healthy controls. Cervical extensor muscle stiffness on the headache side was higher than that on the non-headache side and in healthy controls. *p < 0.05 and **p < 0.01. TRAP, trapezius; SPL, splenius capitis; SCap, semispinalis capitis; SCer, semispinalis cervicis.

Increased stiffness could be observed in the superficial cervical extensor muscles of patients with CEH. The stiffness of all muscles examined was higher in the headache side compared to the non-headache side and healthy controls. Significantly increased stiffness was found in TRAP, SCap, and SCer in the headache side compared to the non-headache side in patients with CEH (*p* < 0.001). Compared to healthy controls, the stiffness of TRAP, SPL, SCap, and SCer was significantly increased in the headache side of patients with CEH (*p* < 0.01). Significantly increased stiffness in SCer was found on the non-headache side of patients with CEH compared to healthy controls (*p* < 0.01) (Figure 2 and Supplementary Table S1). In addition, superficial cervical extensor muscle stiffness gradually increased from superficial to deep in the two groups, with the lowest TRAP stiffness and the highest SCer stiffness.

### Correlation Between Stiffness and VAS, Age, and BMI

**Figure 3:**
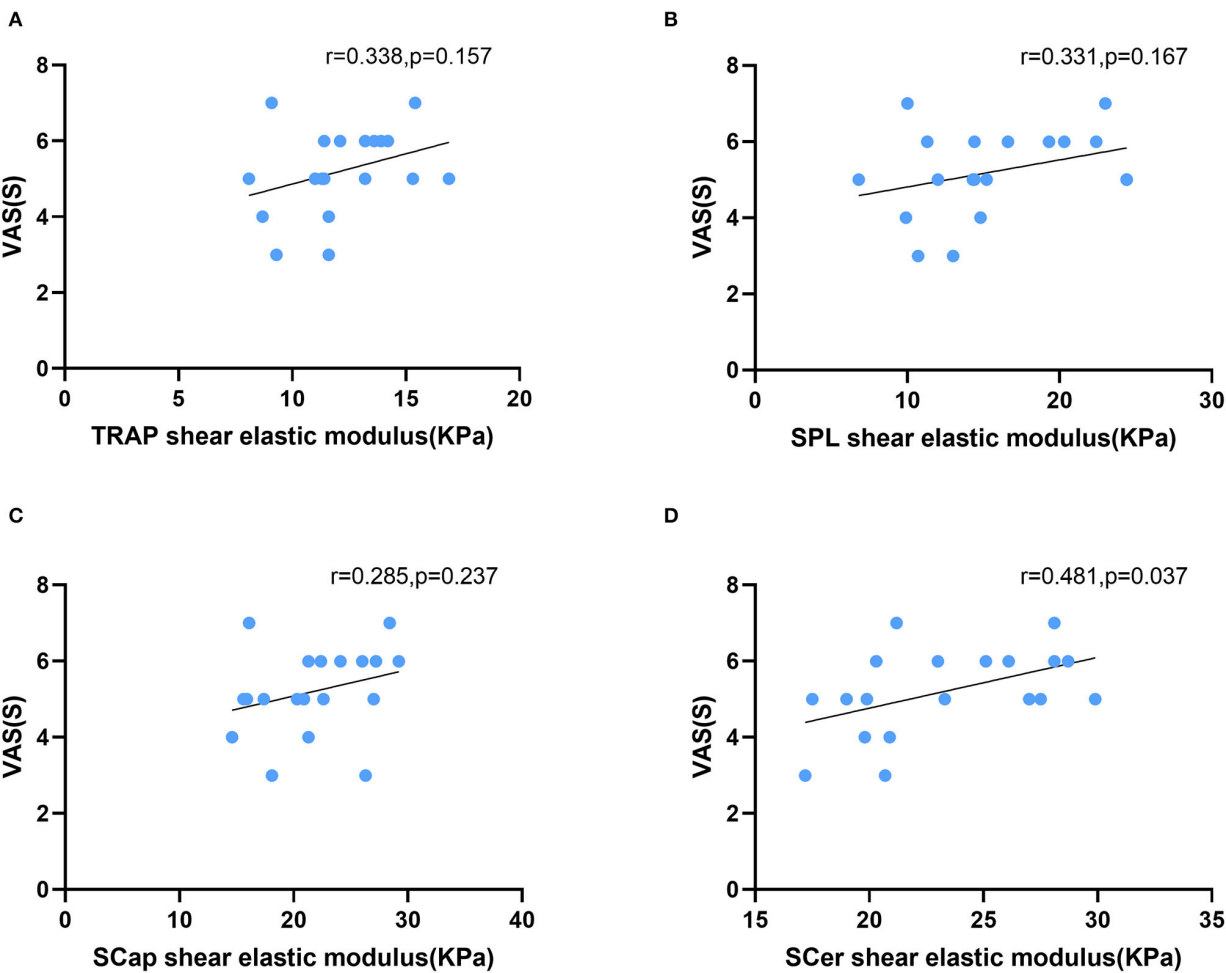
Correlation between superficial cervical extensor muscle stiffness and VAS scores in patients with CEH. The stiffness of SCer **(D)** was positively correlated with VAS scores. The stiffness of TRAP **(A)**, SPL **(B)**, and SCap **(C)** was not correlated with VAS scores. TRAP, trapezius; SPL, splenius capitis; SCap, semispinalis capitis; SCer, semispinalis cervicis.

In patients with CEH, headache side stiffness and VAS scores were positively correlated in SCer (*r* = 0.481, *p* = 0.037). No correlation was found between stiffness and VAS in TRAP (*r* = 0.338, *p* = 0.157), SPL (*r* = 0.285, *p* = 0.237), or SCap (*r* = 0.183, *p* = 0.455) (Figure 3). There was no correlation between stiffness and age in TRAP (*r* = −0.185, *p* = 0.449), SPL (*r* = −0.001, *p* = 0.998), SCap (*r* = −0.066, *p* = 0.789), or SCer (*r* = −0.186, *p* = 0.445). No correlation was found between stiffness and BMI in TRAP (*r* = −0.190, *p* = 0.437), SPL (*r* = −0.259, *p* = 0.284), SCap (*r* = −0.083, *p* = 0.735), or SCer (*r* = −0.305, *p* = 0.205). Similarly, no correlation was found between superficial cervical extensor muscle stiffness on the non-headache side and VAS, age, or BMI.

### ROC Curve Analysis

**Figure 4:**
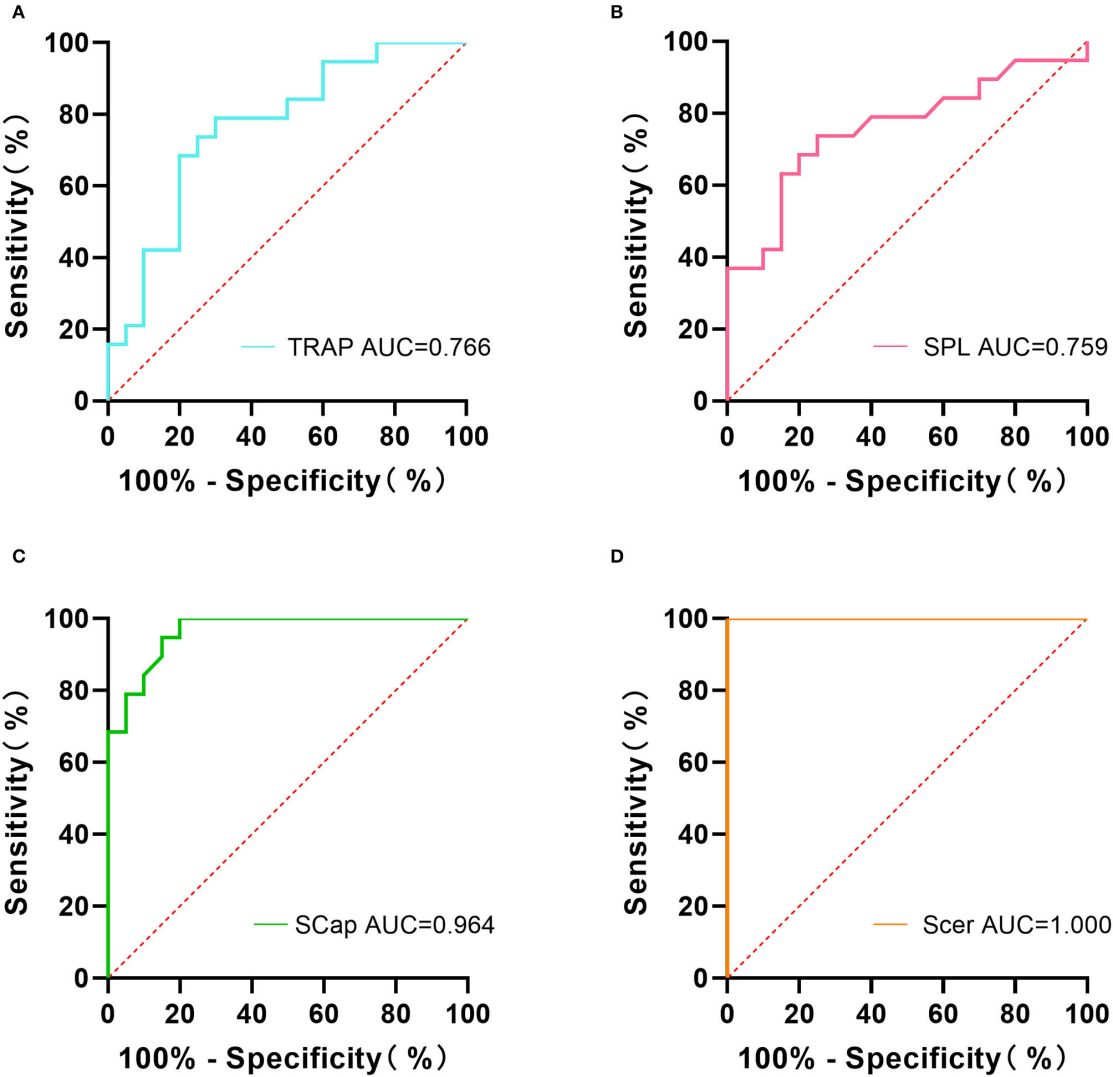
Receiver operating characteristic (ROC) analysis of superficial cervical extensor muscle stiffness in diagnosing CEH. The AUC values of TRAP **(A)**, SPL **(B)**, SCap **(C)**, and SCer **(D)** were 0.766, 0.759, 0.964, and 1.000, respectively. AUC, area under the curve; TRAP, trapezius; SPL, splenius capitis; SCap, semispinalis capitis; SCer, semispinalis cervicis.

The ROC curve analysis showed that the area under the curve (AUC) of TRAP, SPL, SCap, and SCer were 0.766, 0.759, 0.964, and 1.000, respectively (Figure 4). The sensitivity and specificity of TRAP stiffness were 78.95% and 70.00%, respectively. The sensitivity of SPL was 73.68%, and its specificity was 75.00%. The sensitivity and specificity of SCap stiffness were 100% and 80.00%, respectively. The sensitivity and specificity of SCer were up to 100.00% (Table 2). The results indicated that combined sensitivity and specificity of superficial cervical extensor muscle stiffness are fair, and the stiffness of SCap and SCer had a higher diagnostic ability for CEH.

**Table 2:** The diagnostic ability of superficial cervical extensor muscle stiffness for CEH.

	**TRAP**	**SPL**	**Scap**	**Scer**
AUC	0.766	0.759	0.964	1.000
95%CI	0.615–0.916	0.603–0.916	0.916–1.000	1.000–1.000
*P*-value	<0.01	<0.01	<0.0001	<0.0001
Sensitivity	78.95%	73.68%	100.00%	100.00%
Specificity	70.00%	75.00%	80.00%	100.00%

*TRAP, trapezius; SPL, splenius capitis; SCap, semispinalis capitis; SCer, semispinalis cervicis; AUC, area under the curve; CI, confidence interval*.

*p < 0.05 was considered significant*.

## Discussion

In this cross-sectional study, we found that the stiffness of all the examined superficial cervical extensor muscles was higher on the headache side of patients with CEH than on the non-headache side and in healthy controls. The stiffness of SCer on the non-headache side of patients with CEH was also increased compared to healthy controls. In patients with CEH, the stiffness of SCer was positively correlated with VAS scores. There was no correlation between the stiffness of lateral TRAP, SPL, SCap, SCer, and age or BMI. Measured muscle stiffness had fair combined sensitivity and specificity in the diagnosis of CEH, and the stiffness of SCer had the best diagnostic ability to distinguish patients with CEH from healthy controls. The results were consistent with the manifestations of cervical lesions and their soft tissues in patients with CEH. Our study provided evidence that superficial cervical extensor muscle stiffness was increased in patients with CEH, with data supporting the clinical findings of increased cervical muscle stiffness in patients with CEH.

Neck muscle tension and stiffness were common symptoms of patients with CEH (8), and pain in the back of the neck generally appeared earlier than in the head (25). The TRAP tension of patients with CEH was significantly increased relative to that of healthy controls. A recent study found that patients with CEH had increased TRAP tension and stiffness during the resting state and muscle contraction state, but their stiffness was not statistically different from that of healthy controls (26). The results were different from our results, possibly due to the inconsistent location of TRAP measurements (Seung et al. measured near C7 and we measured near C4). Karadas et al. injected 15 U of botulinum toxin type-A into the SPL of CEH-affected side (treatment group) and 0.2 ml of normal saline in the placebo group. After 12 weeks of treatment, VAS scores and headache frequency in the treatment group were significantly lower than those in the pretreatment and placebo group (27). Botulinum toxin was mainly used to treat muscle stiffness, spasms, and dystonia (28). In this study, we also found increased stiffness of SPL on the affected side of patients with CEH. The headache of patients with CEH may be alleviated by reducing SPL stiffness. However, more clinical trials are needed to confirm this hypothesis. Sureeporn et al. (29) used magnetic resonance imaging (MRI) to study the pathological changes of cervical muscles in elder women with CEH and found that patients with CEH had more fat infiltration in the affected TRAP and SCap compared to healthy subjects. Jull et al. (3) used ultrasound to evaluate cervical muscle changes in patients with CEH and also found varying degrees of fat infiltration in TRAP and SCap. Fat infiltration may decrease shear elastic modulus values (30). As the disease progresses, muscle fibers decrease, become thinner or even disappear, and the extracellular matrix, such as elastin, stores an insufficient amount of energy, which may increase muscle stiffness (31, 32). Our study found increased stiffness in SPL and SCap of patients with CEH, which was also observed in patients with migraine (33). SPL and SCap stiffness may be one of the important factors associated with the onset of CEH and migraine. SCer was the deep muscle of the cervical vertebra region and consists mainly of slow muscle fibers with a low threshold (34). Slow muscle fibers are mainly type I fibers. *In vitro* animal studies showed that type I fibers were harder than type II fibers (35). Researchers have shown that the deep muscle around the cervical vertebra was more saturated with fat than the superficial muscle (36, 37). These can partly explain the finding in this study that superficial cervical extensor muscle stiffness gradually increased from superficial to deep. The internal structure of the superficial cervical extensor muscles in patients with CEH is changed, and the physical property of elasticity may also be changed. Our results showed that superficial cervical extensor muscle stiffness on the headache side of patients with CEH was significantly greater than that on the non-headache side and in healthy controls.

Pain correlates with the biomechanical properties of the muscles (38). Muscles in the painful area of the body are often accompanied by increased stiffness (39). Increased stiffness may be explained by insufficient elastin storage energy or impaired elastic fiber function due to faulty cell signaling mediated by microfibrils (40). Elastin and fibrillin microfibrils are important components of elastic fibers. In this study, we found a positive correlation between the stiffness of the affected Scer and VAS scores, suggesting that headache severity was related to SCer stiffness. A study on chronic low back pain supported the finding that VAS scores were positively correlated with the stiffness of the affected muscle (38). However, no correlation was found between the stiffness of TRAP, SPL, and SCap, and VAS scores in our study, which may be because Scer, as a deep muscle, has a greater supporting and stabilizing effect on the cervical vertebra. Wu et al. (41) reported that muscle stiffness gradually decreases with age, but the association was not found in our study, possibly because CEH alters muscle stiffness. In addition, our results were consistent with a study that investigated low back pain. Shane et al. (42) investigated the correlation between demographics and muscle stiffness in patients with back pain, and no correlation was found between affected muscle stiffness and age or BMI.

Currently, the diagnosis of CEH depends mainly on clinical manifestations, without specific imaging or laboratory examination, and the clinical manifestations overlap with tension-type headache (TTH) and migraine, making them difficult to distinguish. It has been found that the stiffness of TRAP, SPL, SCap is significantly greater in patients with TTH and migraine than in healthy controls (33, 43), but the diagnostic ability of cervical extensor muscle stiffness in patients with TTH and migraine is not clear. Our study showed that the stiffness of TRAP (AUC = 0.766), SPL (AUC = 0.759), SCap (AUC = 0.964), and SCer (AUC = 1.000) could effectively distinguish CEH from healthy controls, among which SCer stiffness had the strongest diagnostic ability for CEH.

Our study has several limitations. Firstly, although SWE is performed at a specific vertebra level and in a standard position from the spinous process, intramuscular variations may make the data to have a small extent of variability (23, 44). Secondly, only patients with CEH with unilateral headache were included, and changes in superficial cervical extensor muscle stiffness in patients with bilateral headache are unknown. In addition, the study used the ICHD-3 diagnostic criterion, which is restrictive with respect to cervical spine lesions for the diagnosis of CEH. Some patients with CEH might not have an obvious cervical disorder and were not included in this study. Finally, headache symptoms in patients with CEH, such as the frequency or duration of headache, were not fully evaluated. Due to the limited sample size, the results of our study need to be validated in a larger cohort.

In conclusion, our study found significantly increased stiffness in the superficial cervical extensor muscles of patients with CEH. The stiffness of the superficial cervical extensor muscles, especially SCer, has a fair diagnostic ability for CEH. Whether the stiffness of the superficial cervical extensor muscles is related to the severity of CEH remains to be studied.

## Data Availability Statement

The raw data supporting the conclusions of this article will be made available by the authors, without undue reservation.

## Ethics Statement

The studies involving human participants were reviewed and approved by Medical Ethics Committee of Weifang People's Hospital. The patients/participants provided their written informed consent to participate in this study.

## Author Contributions

LZL, YNY, SYZ, XZY, and JCF developed the study concept and design. LZL, YNY, and JCF collected clinical and imaging data and analyzed and interpreted the data. PWG, CFX, and XG analyzed the data. SYZ and XZY designed and revised this manuscript. LZL wrote the first draft. All authors critically reviewed this manuscript, contributed to this article, and approved the submitted version.

## Funding

This study was supported by grants from the Weifang Health Commission Fund Project (wfwsjk-2021–161).

## Conflict of Interest

The authors declare that the research was conducted in the absence of any commercial or financial relationships that could be construed as a potential conflict of interest.

## Publisher's Note

All claims expressed in this article are solely those of the authors and do not necessarily represent those of their affiliated organizations, or those of the publisher, the editors and the reviewers. Any product that may be evaluated in this article, or claim that may be made by its manufacturer, is not guaranteed or endorsed by the publisher.
