# Supplementary material for: Increased Stiffness of the Superficial Cervical Extensor Muscles in Patients With Cervicogenic Headache: A Study Using Shear Wave Elastography
Source: Front Neurol. 2022 May 27;13:874643. doi: 10.3389/fneur.2022.874643 (PMC9184726; doi:10.3389/fneur.2022.874643)
Supplement: Supplementary file 1 [file Table_1.DOCX]

## Supplementary Materials


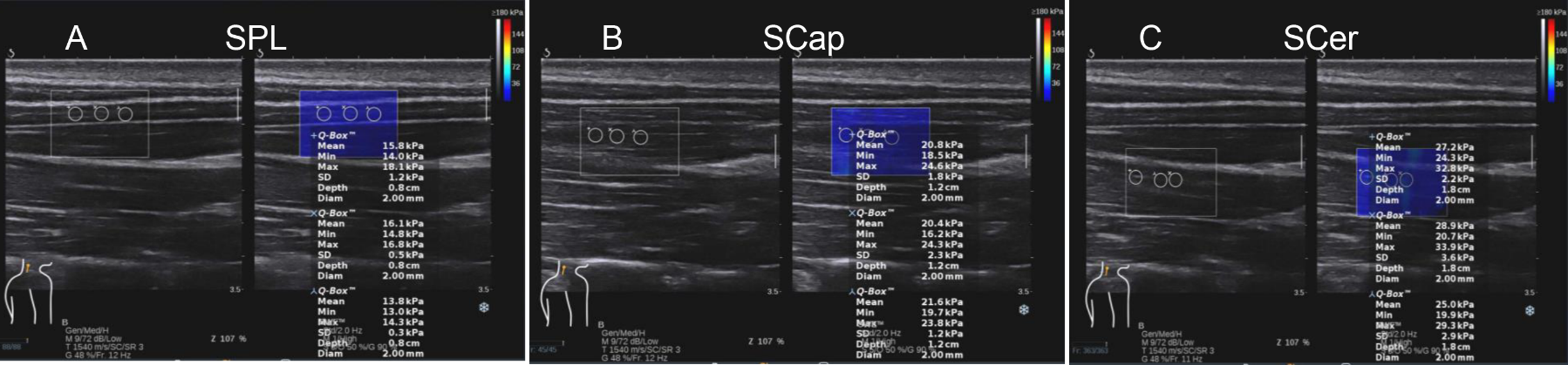


**Figure S1** Example images showing shear elastic modulus of SPL, SCap, and SCer in CEH patients. The stiffness of three ROIs in SPL (**A**) are 15.8 KPa, 16.1 KPa, and 13.8 KPa, respectively. The stiffness of three ROIs in SCap (**B**) are 20.8 KPa, 20.4 KPa, and 21.6 KPa, respectively. The stiffness of three ROIs in SCer (**C**) are 27.2 KPa, 28.9 KPa, and 25.0 KPa, respectively. SPL, splenius capitis; SCap, semispinalis capitis; SCer, semispinalis cervicis.

**Table S1** Comparison of superficial cervical extensor stiffness

|  | Headache side（KPa） | Non-headache side（KPa） | Healthy controls（KPa） |
| --- | --- | --- | --- |
| TRAP | 12.17$\pm$2.41#* | 10.57$\pm$2.55 | 9.92$\pm$2.22 |
| SPL | 15.64$\pm$5.28# | 12.54$\pm$3.43 | 11.31$\pm$2.21 |
| SCap | 21.83$\pm$4.67#** | 14.34$\pm$2.67 | 13.15$\pm$2.31 |
| SCer | 23.33$\pm$4.08#** | 16.32$\pm$2.77# | 12.77$\pm$1.74 |

*P*<0.05 was considered significant. Comparison of superficial cervical extensor stiffness between healthy controls and CEH patients: # indicates *P*$<$0.01. Comparison between headache side and non-headache side in CEH patients: * indicates *P*$<$0.05 and ** indicates *P*<0.01. TRAP, trapezius; SPL, splenius capitis; SCap, semispinalis capitis; SCer, semispinalis cervicis; CEH, cervicogenic headache.
